# Acrylamide Decreases Cell Viability, and Provides Oxidative Stress, DNA Damage, and Apoptosis in Human Colon Adenocarcinoma Cell Line Caco-2

**DOI:** 10.3390/molecules25020368

**Published:** 2020-01-16

**Authors:** Adriana Nowak, Małgorzata Zakłos-Szyda, Dorota Żyżelewicz, Agnieszka Koszucka, Ilona Motyl

**Affiliations:** 1 Department of Environmental Biotechnology, Faculty of Biotechnology and Food Sciences, Lodz University of Technology, Wólczańska 171/173, 90-924 Łódź, Poland; agnieszka.koszucka@gmail.com (A.K.); ilona.motyl@p.lodz.pl (I.M.); 2 Institute of Molecular and Industrial Biotechnology, Faculty of Biotechnology and Food Sciences, Lodz University of Technology, Stefanowskiego 4/10, 90-924 Łódź, Poland; malgorzata.zaklos-szyda@p.lodz.pl; 3 Institute of Food Technology and Analysis, Faculty of Biotechnology and Food Sciences, Lodz University of Technology, Stefanowskiego 4/10, 90-924 Łódź, Poland; dorota.zyzelewicz@p.lodz.pl

**Keywords:** acrylamide, cytotoxicity, genotoxicity, apoptosis/necrosis, ROS generation, oxidative DNA damage, scanning electron microscope, mitochondrial membrane potential

## Abstract

Acrylamide (AA) toxicity remains an interesting subject in toxicological research. The aim of the research performed in this paper was to determine mechanisms of cyto- and genotoxic effects of AA on the human colon adenocarcinoma cell line Caco-2, to estimate the inhibitory concentration (IC)_50_ values in cell viability assays, to measure the basal and oxidative DNA damage as well as the oxidative stress leading to apoptosis, and to assess the morphological changes in cells using microscopic methods. It has been proven that AA induces cytotoxic and genotoxic effects on Caco-2 cells. Higher cytotoxic activity was gained in the 3-(4,5-dimethylthiazol-2-yl)-2,5-diphenyltetrazolium bromide (MTT) assay compared with the PrestoBlue assay, with IC_50_ values of 5.9 and 8.9 mM after 24 h exposure, respectively. In the single-cell gel electrophoresis assay, the greatest DNA damage was caused by the highest concentration of acrylamide equal to 12.5 mM (89.1% ± 0.9%). AA also induced oxidative DNA damage and generated reactive oxygen species (ROS), which was concentration dependent and correlated with the depletion of mitochondrial membrane potential and apoptosis induction. In the microscopic staining of cells, AA in the dosage close to the IC_50_ induced morphological changes typical for apoptosis. Taken together, these results demonstrate that AA has a pro-oxidative effect on Caco-2 cells, leading to apoptotic cell death.

## 1. Introduction

It has been known for a long time that the formation of certain chemicals during food processing or preparation may pose a risk to human health. Acrylamide (AA) is a synthetic chemical widely used in various industries. Moreover, AA also occurs in thermally processed food as a carcinogen—it is formed via the Maillard reaction when heating carbohydrate-rich foods at temperatures above 120 degrees Celsius, especially potatoes and cereal-based products [1,2]. The presence of the compound in food is very substantial, owing to its mutagenic and carcinogenic properties. The International Agency for Research on Cancer (IARC) classified AA as a potentially carcinogenic substance for humans, as it possibly causes DNA damage and gene mutation [3]. It stated that AA is a probable human carcinogen, on the basis of its carcinogenicity in rodents. The results suggest that the compound can increase the incidence of cancers in organs, such as lungs, brain, liver, skin, kidney, thyroid, testes, and breast [4,5,6,7,8]. Hogervorst et al. [7] showed a statistically significant positive association between AA intake and the risk of colorectal tumours. In the human body, AA undergoes conjugation with glutathione, either non-enzymatically or by glutathione-S-transferases via an epoxidation reaction mediated by cytochrome P-450 CYP2E1 [9]. The metabolite of that reaction is glycidamide. Both AA and glycidamide can be bound to haemoglobin, albumins, proteins, and DNA; however, glycidamide is more reactive than AA and is considered to be more genotoxic [6,9]. Many studies suggest that AA could be a potent neurotoxic agent, and that long-term exposure could lead to damage of both the peripheral and central nervous systems [10]. It is also responsible for the progression of atherosclerosis [11].

Previous toxicological studies have indicated that AA is associated with carcinogenesis, neurotoxicity, genotoxicity, and reproductive toxicity. Recently, the European Food Safety Authority (EFSA) announced that developmental toxicity is probably a critical endpoint for the hazard identification and characterization of AA, with the consideration that infants, toddlers, and other children are the most susceptible exposed groups regarding chronic dietary AA in the human exposure estimation [12]. It has been demonstrated in many countries that a wide range of population groups, ranging from infants to elderly, are exposed to dietary AA, which is directly related to the AA content in the food consumed; that is, there can be even up to 5849 µg of AA per kg of biscuits, up to 4180 µg/kg of potato crisps, and up to 7095 µg/kg of coffee (which equals approximately 101 µM/kg) [13]. According to recent data, the average human intake is estimated to be 0.6 μg/kg body weight/day for the general adult population. However, it was shown that, for high consumers, intakes were up to 4 μg/kg body weight/day [14]. Accordingly, on the basis of the average body weight of 70 kg, the daily intake of AA could achieve a total dosage of 14 to 70 μg/day for adults, while for children, the total dosage could range from 70 to 280 μg/day. Selected legal regulations in the European Union concerning AA content in foodstuffs were discussed previously in our recent review article [15]. It is worthwhile to mention that AA can be also present in water, cigarette smoke, food packaging, some adhesives, textiles, cosmetics, plastics, and agricultural industry. Thus, the cyto-, geno-, and neurotoxic potential of AA in food and its impact on cancer risk in humans is of great concern. Despite the many epidemiological and toxicological studies regarding AA, there is not enough evidence to indicate that the amounts of AA consumed in everyday diet can result in cancer [16]. However, it is worth noticing that these determinations are based on studies on laboratory animals, not on people’s exposure to AA from foods. Further, the effect of food mutagens in carcinogenesis can be modified by heritable traits, namely, low-penetrant genes that affect mutagen exposure of DNA through metabolic activation and detoxification or cellular responses to DNA damage through DNA repair mechanisms or cell death [17].

Despite a large interest regarding AA in the scientific community, there remains an insufficient amount of studies concerning the cytotoxicity and genotoxicity of this compound on cell lines, although this situation has started to change over the last few years. There is one brief piece of research regarding AA genotoxicity, which was conducted on the human colon adenocarcinoma cell line Caco-2 and normal V79 fibroblasts to compare AA with the genotoxicity of glycidamide [9]. Three more research papers concern AA cytotoxicity and oxidative stress reduction by myricitrin [18], olive oil hydroxytyrosol [19], and jackfruit flake after in vitro digestion [20]. In light of the above, the aim of this study was to assess the comprehensive mechanisms of the cytotoxic and genotoxic action of AA on Caco-2 cell line. That cell line is used as an in vitro human model of epithelial cells originating from the gastrointestinal tract, which corresponds to the primary target tissue subjected to AA in vivo. The biological activities of cells treated with AA were examined with the application of various cellular and morphological methods in regards to metabolic activity, genotoxicity, mitochondrial membrane potential, intracellular oxidative stress generation, and apoptosis induction (morphological and biochemical observations).

## 2. Results and Discussion

### 2.1. Effect of AA Treatment on Cell Proliferation

**Figure 1:**
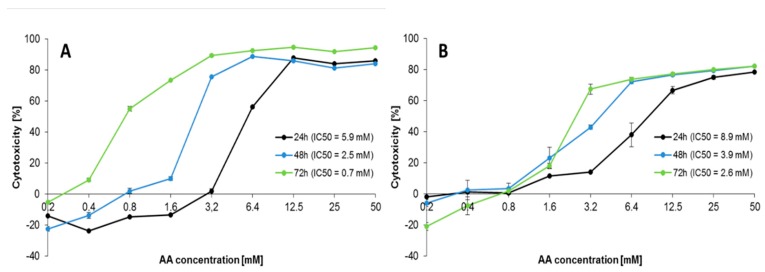
Caco-2 cells proliferation in the presence of acrylamide after 24–72 h exposure; measured by the (**A**) 3-(4,5-dimethylthiazol-2-yl)-2,5-diphenyltetrazolium bromide (MTT) and (**B**) PrestoBlue assays. Each data point represents the mean of the absorbance/fluorescence values from cells from eight individual wells. Results are presented as mean ± standard deviation (SD)/ standard error of the mean (SEM), respectively. IC, inhibitory concentration.

Treatment of Caco-2 cells with AA (0.2–50 mM) resulted in a time- and dose-dependent decrease in cell viability, as measured by both MTT and PrestoBlue assays after 24–72 h (Tables S1 and S2, Supplementary Materials). Statistics for MTT and PrestoBlue assays are presented in Tables S1 and S2. Both assays showed that different concentrations of AA treatment significantly decreased Caco-2 cell proliferation at 24–72 h when compared with the AA unexposed negative control (*p* < 0.05). The correlations between the AA concentration and cell proliferation were presented in Figure 1A,B. In the presence of the highest AA concentration (50 mM), cytotoxicity exceeded 84.0%–94.4% and 78.4%–82.2% after 24–72 h exposure in MTT and PrestoBlue assays, respectively. Exposure to 6.4–50 mM of AA (24 h), 3.2–50 mM of AA (48 h), and 0.8–50 mM of AA (72 h) showed a significant increase in AA cytotoxicity in the MTT assay, while in the PrestoBlue assay, AA induced cytotoxic effects from 6.4–50 mM of AA (24 h) and 1.6–50 mM of AA (48–72 h) (*p* < 0.05). The inhibitory concentration (IC)_50_ values after 24–72 h of exposure to AA showed higher cytotoxicity in the MTT assay (5.9, 2.5, and 0.7 mM) than PrestoBlue assay (8.9, 3.9, and 2.6 mM), respectively. Different values obtained for each of the assay types resulted from the diverse molecular mechanism used by them. Despite the fact that they are used for quantitative measurements of products generated by mitochondrial and cytosol dehydrogenases, the different structures of the substrates strongly determined the region the reaction occurred and the assay sensitivities [21]. MTT is reduced inside the cells to insoluble formazan, while the resazurin-based PrestoBlue reagent present in the culture medium can be reduced by mitochondrial reductases and other cellular enzymes. In contrast to the resazurin-based reduction signifying a disturbance of cellular metabolism, the tetrazolium–salt substrate also reacts when interruption to electron transport and mitochondrial dysfunction occurs. Thus, the higher sensitivity of MTT may result from AA influence on cellular mitochondria, causing an additional positive effect to the disturbed metabolism in cells. Despite this, our results are in accordance with another study performed with metabolic activity-based assays, however, owing to the fact that the different cellular models and tissue origins influenced the different sensitivities of used cells to AA, the IC_50_ values also varied. Chen et al. [18], in their study on the inhibition of AA cytotoxicity on Caco-2 cells in MTT assay by myricitrin—a naturally occurring flavonoid derived from Chinese bayberry bark and fruit—demonstrated an IC_50_ value of AA close to 5 mM after 48 h exposure. The IC_50_ of AA for 24 h exposure of NIH/3T3 fibroblasts was 6.73 mM as estimated by MTT assay [22]. For the adenocarcinoma alveolar-basal epithelial cells A549, the IC_50_ after 24 h was 4.6 mM [23], and for the normal human lung epithelial cells BEAS-2B, it was 2.0 mM [24]. The cytotoxic and antiproliferative activity of AA was demonstrated by some authors for several cancer and normal cell lines (e.g., human neuroblastoma SH-SY5Y; human astrocytoma U-1240 MG; neural progenitor cell line C17.2; murine microglial cell line BV2; A549; NIH/3T3 fibroblasts; cervical cancer HeLa [22,23,24,25,26,27,28]). According to Kacar et al. [24], AA interferes with kinesin proteins, which are responsible for the spindle formation during cell division, thus inhibiting cell proliferation. Mechanisms of AA toxicity were the subject of profound reviews [14,29]. In the subsequent analysis, we wanted to detect mechanisms of AA toxicity in the Caco-2 cell line. The obtained data allowed as to choose appropriate concentrations of AA for further investigations.

### 2.2. Effect of AA Treatment on Basal and Oxidative DNA Damage

**Figure 2:**
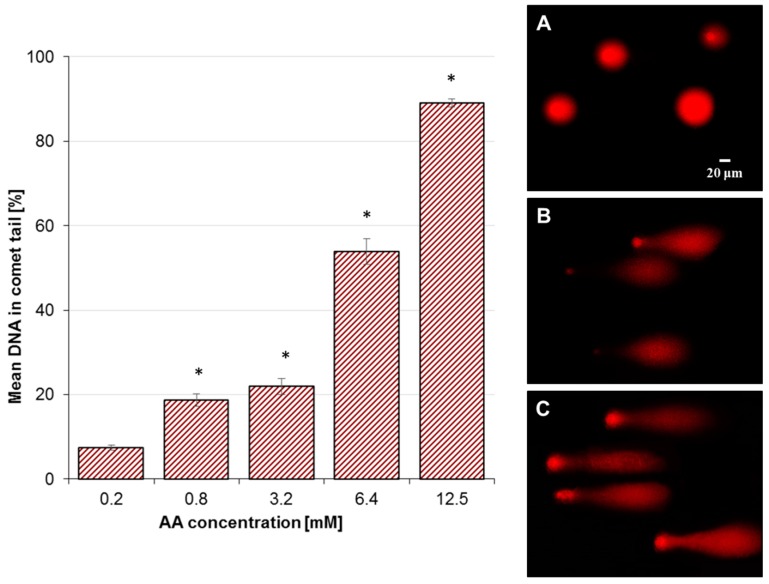
Basic DNA damage in Caco-2 cells after exposure to acrylamide, expressed as the mean percentage of DNA in the comet tail in the alkaline comet assay. One hundred cells were analyzed for each treatment. Results are presented as mean ± standard error of the mean (SEM). * Results are significantly different from the unexposed control, analysis of variance (ANOVA) (*p* < 0.05). (**A**–**C**) Representative images of 1 mg/mL PI-stained comets: (**A**) untreated cells; (**B**) cells treated with 6.4 mM acrylamide; (**C**) positive control (50 μM H_2_O_2_). Fluorescence microscopy; 200× magnification.

As the test concentration of the substance for the comet assay should range from the maximum acceptable cytotoxicity to little or no cytotoxicity (because DNA damage is associated with cell death), the final concentrations of AA selected for genotoxicity assessment were (owing to the IC_50_ values) as follows: 0.2, 0.8, 3.2, 6.4, and 12.5 mM. DNA damage of Caco-2 cells treated with AA increased in a dose-dependent manner (*p* < 0.05) from 7.4% ± 0.6% (0.2 mM) up to 89.1% ± 0.9% (12.5 mM) (Figure 2). So, it was demonstrated that AA induced DNA damage at little or non-cytotoxic concentrations (0.78 mM). The negative control (unexposed Caco-2 cells) yielded a tail DNA of 5.3% ± 0.5% and cell treatment with 50 μM H_2_O_2_ (positive control) resulted in 88.5% ± 1.4% damage (data not shown). The comet tails of Caco-2 cells, after exposure to AA, were analyzed by fluorescence microscope using propidium iodide (PI), as shown in Figure 2A–C. A significant amount of DNA breaks (*p* < 0.05) were induced by AA, as evidenced by the long tails formed by DNA fragmentation. The intensity of the comet tails relative to the head reflects the number of DNA breaks. The results are important when regarding the possibility that genotoxicity may lead to cancer-causing mutations.

**Figure 3:**
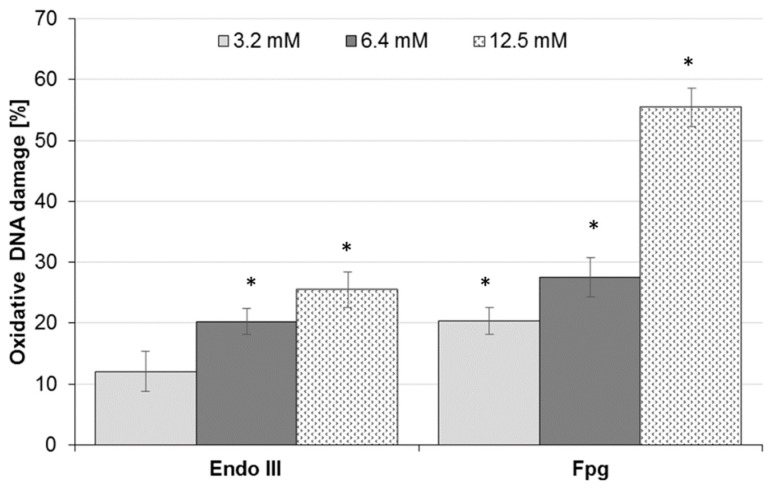
Oxidative DNA damage measured as the mean comet tail of Caco-2 cells following exposure to acrylamide (AA) recognized by endonuclease III (Endo III) or formamidopyrimidine-DNA glycosylase (Fpg). Fifty cells were analyzed for each treatment. Results are presented as mean ± standard error of the mean (SEM). * Results are significantly different from unexposed control, ANOVA (*p* < 0.05).

In order to understand the mechanism involved in DNA damage, a comet assay was also performed in Caco-2 cells exposed to AA in the presence of repair enzymes, endonuclease III (Endo III), which detects oxidized pyrimidines, and Fpg, which recognizes the common oxidized purine 8-OHgua (7,8-dihydro-8-oxo-guanine). The model of the research has not yet been published for Caco-2 cells. The greatest extent of DNA oxidation was recognized by Fpg for cells treated with 12.5 mM AA, calculated as 55.4% ± 3.1% (*p* < 0.05) (Figure 3). For the positive control treated with 50 µM H_2_O_2_, the oxidative DNA damage was equal to 51.8% ± 3.5% and 49.4% ± 3.6%, as recognized by Endo III and Fpg, respectively (data not shown). Non-exposed Caco-2 cells (negative control) exhibited little DNA damage, at 0.7% ± 2.1% and 0.8% ± 2.5% for Endo III and Fpg, respectively (data not shown). The results revealed an increase in oxidative DNA damage in Caco-2 cells treated with AA in a dose-dependent manner, yielding between 12.1% ± 3.3% and 25.4% ± 3.0%, and between 20.4% ± 2.2% and 55.4% ± 3.1%, for Endo III and Fpg enzymes, respectively (Figure 3). Overall, the oxidative damage recognized by Fpg was greater than by Endo III across all AA concentrations. Moreover, for 12.5 mM AA, the percentage of detected oxidative damage was more than two times higher for oxidized purine 8-OHgua by Fpg of 55.4% ± 3.1% than for oxidized pyrimidines by Endo III of 25.4% ± 3.0%.

In the research on genotoxicity of AA, it was demonstrated that a prolonged exposure to AA induced gene mutations and chromosomal aberrations, sister chromatid exchanges, micronuclei, polyploids, and aneuploids [10,30], as well as covalent adducts with protaminase in the germ cells of mice in vivo [31]. Some research shows the capacity of AA to induce DNA damage in the comet assay in human normal lymphocytes, human hepatoma G2 (HepG2), and rat adrenal gland cells PC12 [32,33,34]. It compromises DNA repairs damaged by hydrogen peroxide and increases caspase-3 activity, leading to apoptosis. Furthermore, reactive oxygen species (ROS) can be generated by AA, leading to DNA breaks [23,32,35]. Puppel et al. [9] demonstrated no capacity of AA to induce DNA breaks in Caco-2 cells—no genotoxicity was induced by 6 mM of AA (DNA damage 5%)—while our research demonstrated that 6.4 mM of AA caused strong DNA damage (above 50%). The differences probably result from different methodologies. The authors applied cells in monolayer, not in suspension, which is more sensitive to chemical treatment than cells growing in monolayer. Comet assay is suitable for both suspension and monolayer cells, however, in a standard version, it is performed on cells detached from the surface, and in genotoxicity assays, the exposure must not be too long to avoid overwhelming the genotoxic effect by its cytotoxic counterpart. Shimamura and co-workers [36] checked the genotoxicity of AA using rat liver, kidney, and brain. Experiments showed that AA induced a significant increase in the number of micronucleated polychromatic erythrocytes in the bone marrow (*p* < 0.01 vs. control), which is correlated with DNA damage [36]. In El-Bohi et al. [37], AA induced single strand breaks in rat hepatocytes, and the increase in genotoxicity was dose dependent.

### 2.3. Effect of AA on Mitochondrial Membrane Potential (MMP) and Oxidative Stress

**Figure 4:**
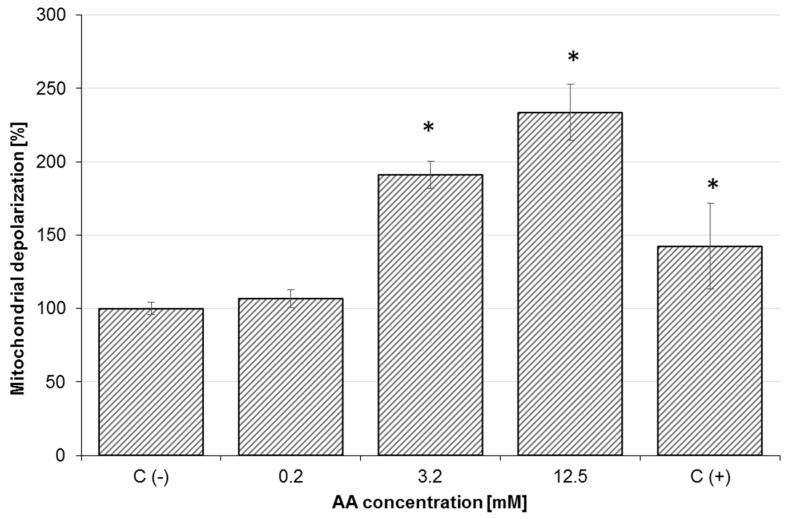
Effect of acrylamide on mitochondrial membrane potential (MMP) depletion in Caco-2 cells after 24 h exposure. C(+)-positive control (50 µM carbonyl cyanide m-chlorophenylhydrazone (CCCP)). Each data point represents the mean of the fluorescence values from cells from four individual wells. Results are presented as mean ± standard error of the mean (SEM). * Results are significantly different from unexposed control-C(−), ANOVA (*p* < 0.05).

**Figure 5:**
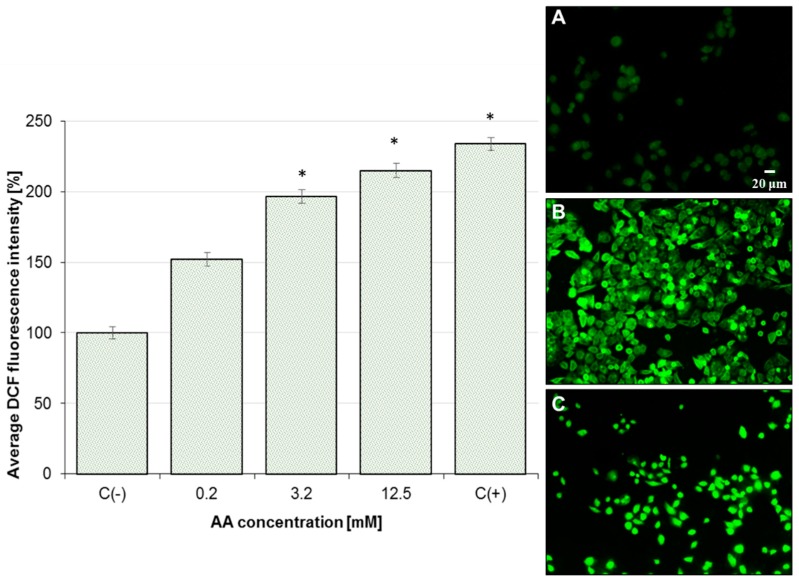
Effect of acrylamide on reactive oxidative species (ROS) generation in Caco-2 cells after 6 h exposure. C(+)-positive control (500 μM H_2_O_2_). Each data point represents the mean of the fluorescence values from cells from five individual wells. Results are presented as mean ± standard error of the mean (SEM). * Results are significantly different from unexposed control-C(−), ANOVA (*p* < 0.05). (**A**–**C**) Representative images of DCFH-DA-stained Caco-2 cells: (**A**) untreated Caco-2 cells; (**B**) Caco-2 cells treated with 3.2 mM acrylamide; (**C**) positive control. Fluorescence microscopy; 200× magnification.

As shown in Figure 4, AA (0.2, 3.2, 6.4, and 12.5 mM) induced a dose-dependent increase in mitochondrial depolarization in Caco-2 cells after 24 h exposure. MMP disruption was greatest in cells treated with 12.5 mM AA, where MMP was 234.0% of the negative control (untreated cells). Reduced mitochondrial potential in Caco-2 cells is associated with increased ROS generation. AA-treated Caco-2 cells resulted in a dose-dependent rise in ROS production (Figure 5) along with oxidative DNA damage (Figure 3). The mean DCF fluorescence of Caco-2 cells was 233.6% of the negative control (untreated cells) after treatment with 12.5 mM AA for 6 h. Therefore, the production of intracellular ROS was analyzed by fluorescence microscope using DCFH-DA, as shown in Figure 5A–C. In AA-treated cells, elevated reactive oxygen species generated via mitochondria impairment oxidized 2,7-dichlorofluorescein, which in turn emitted a bright fluorescence.

The mechanism of acrylamide toxicity begins when there is an imbalance in the biological oxidant to antioxidant ratio, resulting in the potential for oxidative stress to occur. It could also be an initiating step to many diseases. In recent years, it has been demonstrated that AA induces oxidative stress. In research on mice splenocytes performed by Zamani et al. [38], AA affected the cellular redox chain and induced an increase in ROS production, and the peroxidation of lipids and glutathione oxidation, which were accompanied by the collapse of MMP and a reduction in mitochondrial activity. The authors concluded that AA toxicity relied on dysfunction of mitochondria by their oxidative damage, which further seemed to activate cellular death. On the basis of the results from Seydi et al. [39], in male Sprague-Dawley rats, hepatocyte cytotoxicity of AA (1 mM) was mediated by ROS formation and lipid peroxidation. Incubation of hepatocytes with AA resulted in rapid hepatocyte glutathione depletion, which is another marker of the cellular oxidative stress. AA cytotoxicity was also associated with mitochondrial injury, as evidenced by the decline of MMP and lysosomal membrane leakiness. Their results also showed that AA induced caspase-3 activation, the final mediator of apoptosis signalling. These findings contribute to a better understanding mechanism involved in AA hepatotoxicity, originating from oxidative stress and ending in mitochondrial/lysosomal damage and cell death signalling [39].

The ability of antioxidants such as plant extracts, flavonoids, vitamins, or olive oil to prevent AA -induced cytotoxicity, appears to support oxidative stress as the main mechanism of the harmful action of AA [18,40,41,42]. This was discussed in our previous reviews [14,43].

### 2.4. Effect of AA Treatment on Cell Death and Morphology

**Figure 6:**
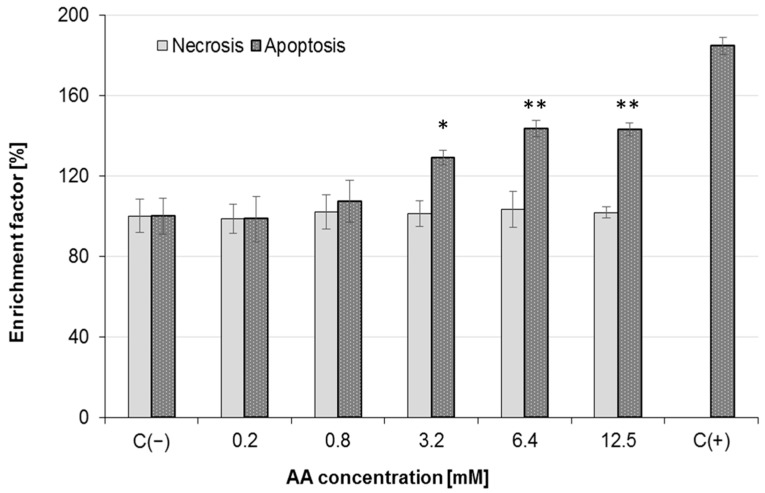
Effect of acrylamide-induced apoptosis and necrosis in Caco-2 cells after 24 h exposure. Each data point represents the mean of the absorbance values from cells from four individual wells. Control cells were not exposed to any compound but the vehicle; results are presented as mean ± standard deviation (SD) from four independent experiments; statistical significance was calculated versus control cells (untreated) at * *p* < 0.05, ** *p* < 0.01.

Owing to the observed intracellular oxidative stress elevation, as well as the mitochondrial depolarization after 24 h exposure to AA, in further experiments, we observed the influence of AA on Caco-2 cell death induction. The results revealed that AA induced apoptosis in Caco-2 cells (Figure 6). Compared with the control cells, which were untreated with AA, a significant elevation of characteristics for apoptosis endonucleases products was detected after the cells’ incubation with AA at a dosage of 3.2 mM. In that case, the level of mono- and oligonucleosomes present in the cytoplasm fraction exceeded the relative value observed in control cells by 30%. After cell incubation with 3.2 mM AA, apoptotic cell death was induced in 30% of the cellular population (*p* < 0.05). At the highest AA dosage (12.5 mM), the enrichment factor exceeded the value of 40%, and thus the apoptosis process was deepened. On the other hand, detection of low molecular weight DNA fragments in the culture medium suggested a necrotic type of cellular death. In that case, DNA fragments were released from the cells as a result of increased cellular membrane permeability and leakage. The obtained results clearly show that AA at the studied concentration ranges was not potent enough to induce necrosis of Caco-2 cells, as the enrichment factor in AA-treated cells was similar in the range of all concentrations studied at 101.4% ± 2.4%, and was not significantly different from unexposed cells. There has been evidence that AA can lead to apoptosis via mitochondrial dysfunction and collapse [23,24,25,40,44]. Sahinturk et al. [22] demonstrated that AA enlarged the activity of caspases 3/7 in NIH/3T3 fibroblasts, which contributed to an increase in early and late apoptotic cells. The caspase 3/7 activities of the AA-treated NIH/3T3 cells were three times greater than those of the untreated NIH/3T3 cells. ROS generation in mitochondria by AA is considered as the main cause of apoptosis induction [23]. An increase in intracellular ROS generation and a collapse in MMP leading to apoptotic cell death seem to be cause of AA toxicity—this was confirmed inter alia in Caco-2 cells [18]; human astrocytoma U-1240 MG cells [25]; rat adrenal gland cells PC12 [45]; rat testis Leydig R2C [46]; and HepG2 cells [40].

**Figure 7:**
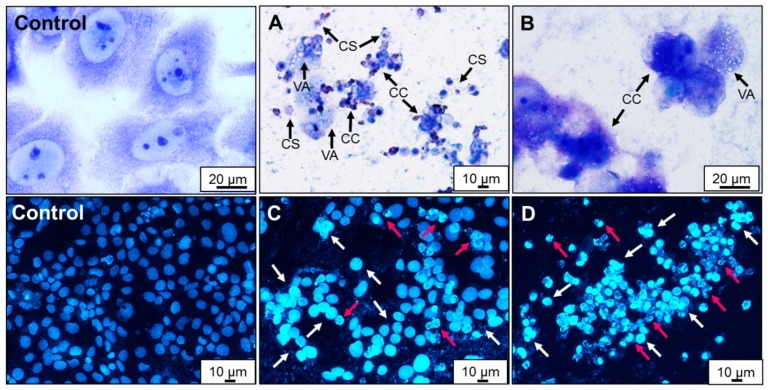
Representative images showing morphology of Caco-2 cells after 24 h exposure to acrylamide. Stained with Giemsa/May–Grünwald: (**A**,**B**) 3.2 mM acrylamide. Chromatin condensation (CC), cell shrinkage (CS), and increased vacuolization (VA). Nuclear morphology of cells after staining with 4′,6-diamidino-2-phenylindole (DAPI): (**C**) 3.2 mM and (**D**) 6.4 mM acrylamide. Condensation of nuclear material (white arrows) and apoptotic bodies (red arrows). Magnifications 200× and 400×.

A big reduction in the Caco-2 cells density as well as an increase in the number of detached and floating cells were clearly observed, especially after incubation of cells with concentrations of AA higher than 12.5 mM. Changes in morphology of cells treated with 3.2 mM AA were observed with the use of Giemsa/May–Grünwald staining (Figure 7A,B). The number of cells per visual field appeared to be fewer than the untreated control, and the monolayer was no longer confluent (Figure 7A,B). Chromatin condensation as well as nuclear abnormalities were observed. Shrinking of the cytoplasm and increased vacuolization occurred. AA induced condensation of vacuoles and cytoplasmic granules. In order to assess nuclear morphology changes induced by AA, Caco-2 cells were stained with DAPI and incubated for 24 h (Figure 7C,D). In the untreated control, cells were spherical, and homogenously stained, and a pattern of normal chromatin was clearly visible (Figure 7). Most AA-treated cells remained detached from the substrate surface (Figure 7C,D). Chromatin condensation and nuclear fragmentation were considered as the main symptoms of apoptosis. In research performed by Sun and co-workers [46], AA caused changes in the morphology of R2C Leydig cells stained with Giemsa stain. R2C cells were fragmented and vacuolated, and the cytoplasm was degenerated (especially at 6 mM AA). In the human astrocytoma cell line U-1240 MG, Chen et al. [25] observed mitochondria with chromatin condensation in vesicular matrix compartments, cytoplasmic vacuole formations were found, and profound mitochondrial swelling and pyknosis were noted, as compared with 0 mM AA treatment.

**Figure 8:**
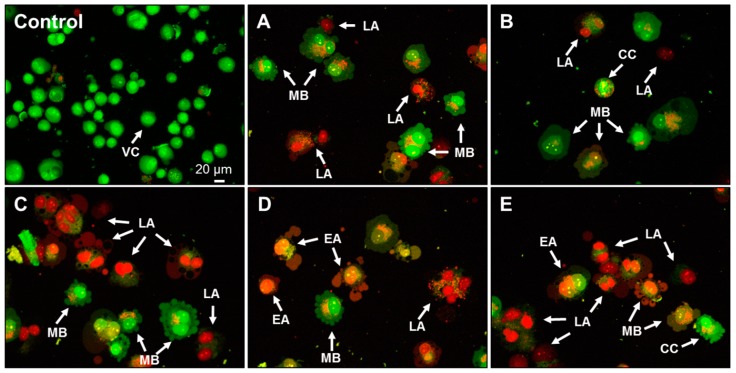
Fluorescent images of acridine orange and propidium iodide (OA/PI)-stained Caco-2 cells after 24 h exposure to (**A**–**B**) 3.2 mM and (**C**–**E**) 6.4 mM acrylamide. Membrane blebbing (MB), chromatin condensation (CC), early (EA) and late apoptosis (LA), and viable cells (VC). Fluorescence microscopy; 200× magnification.

Cells were also stained with acridine orange and propidium iodide (AO/PI), and nuclear changes were observed (Figure 8). AO is an intercalating dye that can permeate both live and dead cells. AO stains all nucleated cells to generate a green fluorescence, while PI can only enter cells with permeable membranes, generating red fluorescence [47]. Condensations of nuclear material and apoptotic bodies of different sizes (indicating late apoptosis) were observed in AA-treated cells (Figure 8A–E). Examples of blebbing and nuclear margination were also found. Noticeable apoptotic morphological changes, including chromatin condensation, confirmed the ability of AA to induce apoptosis in the Caco-2 cell line. Similar morphological changes were observed in A549 lung cells and NIH/3T3 fibroblasts in confocal or transmission electron microscopy analysis [22,24].

**Figure 9:**
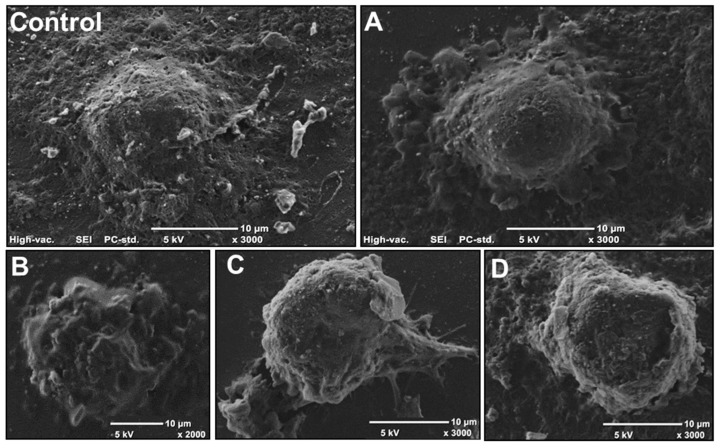
Representative images showing morphology of Caco-2 cells using scanning electron microscope (SEM): (**A**–**D**) cells exposed to 3.2 mM of acrylamide for 24 h; (**A**) membrane and cytoplasm blebbing, higher cell, and cell rounding; (**B**) membrane ruffles; (**C**) membrane and cytoplasm blebbing, higher cell, cell rounding, detaching of the cell, and chromatin condensation; (**D**) membrane and cytoplasm blebbing, higher cell, cell rounding, and chromatin condensation.

Changes in the morphology of Caco-2 cells treated with 3.2 mM AA were demonstrated additionally via scanning electron microscope (SEM) (Figure 9). The control cell (Figure 9A) is shown to be intact and well-preserved to the surface. However, in Figure 9B–D, the cells are clearly rounded and preceding detachment from the substrate surface owing to apoptosis process induction. Caco-2 cell membranes appear to be more porous and irregular owing to the presence of numerous blebs. Membrane and cytoplasm blebbing, as well as chromatin condensation, are also considered as signals for induced apoptosis. Membrane ruffles (Figure 9B) can form as a consequence of inefficient adhesion or cytoskeleton structure disturbance.

Humans are chronically exposed to AA at very low concentrations through diet, especially with the increased consumption of thermally processed carbohydrate rich foods. AA has also been widely used in various industries in soil conditioning, wastewater treatment, cosmetics, paper, textile, irrigation, and drinking water, and has even been found in cigarette smoke [15]. There is only one recommendation, namely that AA exposure must be restricted, either occupationally or via diet. Some cooking and dietary habits should be changed, such as the limitation of frying or roasting, as well as limiting the consumption of foods that might be high in AA. In addition to this, it is important to raise awareness regarding its hazards. In summary, regarding AA daily intake (mentioned in the introduction), the IC_50_ values for that compound seem to be much higher than previously believed to induce cyto- and genotoxicity in cells. Nevertheless, AA possesses strong cyto- and genotoxic potential in Caco-2 cells in vitro, the extent of which depends on its concentration and exposition time. Our work confirms the cytotoxic and genotoxic activity of AA, achieved by decreasing cell viability, and inducing oxidative stress, DNA damage (also oxidative), MMP depletion, and apoptotic cell death induction. We want to emphasize that high AA dosages induced apoptotic-type cell death, which is less destructive than necrosis for neighbouring cells owing to the lack of active enzymes or inflammatory signals released from dying cells. Importantly, our results suggest that AA treatment induces the mitochondria-mediated intrinsic apoptotic pathway. Studies performed on a rat intestinal epithelioid cell line, IEC-6 cells, showed that 5 mM AA induced an overabundance of ROS, leading to a decrease in antioxidant enzyme activities, a subsequent increase in cellular lipids and proteins peroxidation, as well as the depolarization and permeabilization of the mitochondrial membrane [48]. As expected, the release of cytochrome c from the mitochondria to the cytoplasm occurred and a decrease in the Bcl-2/Bax ratio of proteins involved in the apoptosis process was induced. Thus, more detailed evaluations of specific markers connected with cellular death induction by AA in human Caco-2 cells should be performed, such as the activation of caspases or the appearance of specific proteins, that is, t-Bid, cytochrome c, Bax, and Bak. The achieved results contribute to existing research on AA cyto- and genotoxic action in cell lines.

## 3. Materials and Methods

### 3.1. Chemicals and Culture Vessels

Acridine orange (AO), acrylamide (AA), carbonyl cyanide m-chlorophenylhydrazone (CCCP), 4′,6-diamidino-2-phenylindole (DAPI), 2′,7′-dichlorofluorescin diacetate (DCFH-DA), dimethyl sulfoxide (DMSO), 3-(4,5-dimethylthiazol-2-yl)-2,5-diphenyltetrazolium bromide (MTT), Dulbecco’s modified Eagle’s medium (DMEM), ethylenediaminetetraacetic acid (EDTA), Giemsa and May-Grünwald stains, glutaraldehyde, hydrogen peroxide (H_2_O_2_), low melting point (LMP) agarose, mitochondrial membrane potential (MMP) kit, NaCl, NaOH, KOH, KCl, normal melting point (NMP) agarose, propidium iodide (PI), phosphate buffered saline (PBS), streptomycin and penicillin, Triton X-100, Tris, foetal bovine albumin (BSA), and trypan blue were purchased from Sigma-Aldrich (St. Louis, MO, USA). Foetal bovine serum (FBS), HEPES, GlutaMAX^TM^, eight-well Lab-Tek™ chamber slides, PrestoBlue cell viability reagent, and TrypLE^TM^ Express were obtained from Invitrogen, Thermo Fisher Scientific (Waltham, MA, USA). Roux flasks and 6- and 96-well plates were purchased from Becton, Dickinson and Co. (Franklin Lakes, NJ, USA). Cell death detection ELISA^PLUS^ was purchased from Roche Diagnostics (Basel, Switzerland). The human colon adenocarcinoma cell line Caco-2 from the 42nd passage was obtained from Cell Line Service GmbH (Eppelheim, Germany). The 0.22 μm pore size filters were obtained from Merck Millipore (Darmstadt, Germany). Endonuclease III (Endo III) and formamidopyrimidine-DNA glycosylase (Fpg) were obtained from New England Biolabs Inc. (Frankfurt am Main, Germany).

### 3.2. Caco-2 Cell Culture

Caco-2 cells were maintained according to Nowak et al. [49]. The cells were cultured in DMEM supplemented with 10% FBS, 4 mM GlutaMAX^TM^, 25 mM HEPES, 100 µg/mL streptomycin, and 100 IU/mL penicillin. Cells were then incubated in humidifier at 37 °C with 5% CO_2_ for 7–10 days to achieve 80% confluence. The cells were washed every three days with 0.1 M PBS (pH 7.2) and the medium was renewed. Confluent cells were detached from the culture with TrypLE^TM^ Express (37 °C, 10 min) according to the manufacturer’s instruction, then suspended in sterile PBS, and gently aspirated off the plastic flask. The cell suspension was centrifuged (182× *g*, 5 min) and decanted, and then the pellet was re-suspended in fresh DMEM. After performing a cell count by hemocytometer and determining cell viability by trypan blue exclusion, the Caco-2 cells were ready to use.

### 3.3. MTT and PrestoBlue Assays

MTT dye is a yellow tetrazolium, which is reduced to purple formazan in the mitochondria of living cells—the amount of formazan produced is proportional to the activity of the mitochondrial succinate dehydrogenases. PrestoBlue is a resazurin-based membrane permeable solution, which, upon reduction, forms a red fluorescent compound called resorufin via mitochondrial enzymes of viable cells in the tested systems. As a consequence, the reagent exhibits a change in colour, as well as a shift in its fluorescence. For the experiment, 1 × 10^4^ Caco-2 cells were seeded in each well of a 96-well plate in complete culture medium. The cells were incubated overnight at 37 °C under 5% CO_2_. Next, the medium was aspirated and AA in DMEM was added to achieve the final tested concentrations [mM]: 0.2, 0.4, 0.8, 1.6, 3.2, 6.4, 12.5, 25, and 50, added to each well in eight repeats. The negative controls contained only cells in DMEM without AA. Cells were incubated at 37 °C under 5% CO_2_ for 24–72 h. After incubation, the medium with AA was removed from each well and MTT (0.5 mg/mL) was added and incubated at 37 °C under 5% CO_2_ for 3 h. After that time, MTT was removed and formazan precipitates were solubilized by adding DMSO. Absorbance was measured at 550 nm with a reference filter of 620 nm, using a microplate reader (TriStar^2^ LB 942, Berthold Technologies GmbH & Co. KG, Bad Wildbad, Germany). The PrestoBlue assay was performed according to the MTT assay procedure. After incubation, the medium with AA was removed from each well and the PrestoBlue reagent (10% solution in PBS) was added to each well and incubated at 37 °C under 5% CO_2_ for 2 h. Fluorescence was measured at λ_ex_ 560 nm and λ_em_ 590 nm using the above-mentioned microplate reader.

### 3.4. Cytotoxicity and Half Maximal Inhibitory Concentration (IC_50_)

Cytotoxicity and IC_50_ were determined by MTT and PrestoBlue assays according to Nowak et al. [50] and Organisation for Economic Cooperation and Development (OECD) protocol [51]. The absorbance/fluorescence of the control sample (untreated cells) represented 100% cell viability. Cell viability (%) was calculated as follows: [Sample OD or fluorescence/control OD or fluorescence] × 100%], and cytotoxicity (%) was determined as 100 of cell viability (%). The results were presented as mean ± standard deviation (SD)/standard error of the mean (SEM). Experiments were conducted with the same cell population. The value of IC_50_ was determined from curves according to OECD protocol [51].

### 3.5. Genotoxicity Testing (Comet Assay)

Incubation of suspended Caco-2 cells (10^5^ cells/mL) was performed with AA final concentrations (0.2, 0.8, 3.2, 6.4, and 12.5 mM) and without AA as a negative control (1 h at 37 °C). The concentrations were selected based on previous experiments, in which the IC_50_ values of AA were determined. The positive control contained 50 µM H_2_O_2_. All the samples were incubated in DMEM without supplements. The final amount of each sample was set to 1 mL. The comet assay was performed under alkaline conditions (pH > 13) as previously described [50]. After incubation, aliquots of suspended cells were centrifuged (182× *g*, 15 min, 4 °C); decanted; suspended in 0.75% LMP agarose; distributed onto slides precoated with 0.5% NMP agarose; and immersed in lysing solution consisting of 2.5 M NaCl, 1% Triton X-100, 100 mM EDTA, and 10 mM Tris, at pH 10 (4 °C, 1 h). After lysis, DNA was allowed to unwind for 20 min in solution consisting of 300 mM NaOH and 1 mM EDTA. Then, the slides were subjected to horizontal gel electrophoresis in an electrophoretic solution containing 300 mM NaOH and 1 mM EDTA, pH > 13. Electrophoresis was conducted at 4 °C for 30 min at an electric field strength of 0.73 V/cm (300 mA). Then, the slides were neutralized with distilled water. The following day, slides were stained with 1 mg/mL PI and the objects were visualized at 200× magnification with a fluorescence microscope connected to the personal computer-based image analysis system Lucia-Comet v. 7.0 (Laboratory Imaging, Prague, Czech Republic). One hundred images were randomly selected from each sample and the percentage of DNA in the comet tail was measured. The results were presented as mean ± standard error of the mean (SEM).

### 3.6. Oxidative DNA Damage

The procedure was performed according to Nowak et al. [52]. To check the ability of the enzymes Endo III and Fpg to recognize oxidized DNA bases, the cells were incubated either with AA (3.2–12.5 mM) or H_2_O_2_ (50 µM); lysed; and post-treated with Endo III or Fpg, respectively. In short, after lysis in above-mentioned lysing solution, slides were washed three times in an appropriate enzyme buffer (Endo III and Fpg: 40 mM HEPES–KOH, 0.1 KCl, 0.5 mM EDTA, 0.2 mg/mL BSA, pH 8.0), drained, and the agarose was covered with 25 µL of either enzyme buffer or enzyme at 1 µg/mL in buffer, after which it was sealed with a cover glass and incubated for 30 min at 37 °C. Further steps were as described above. To determine the net value of DNA damage recognized by the enzymes, the DNA damage observed in the absence of the enzymes was subtracted from that measured in the presence of them.

### 3.7. MMP Assay

The JC-10 (lipophilic dye) used in that assay forms reversible red-fluorescent in the mitochondria of cells with a polarized mitochondrial membrane. In apoptotic cells, MMP collapse results in the failure to retain JC-10 in the mitochondria, and a return of the dye to its monomeric, green fluorescent form determines the loss of MMP in cells. This test can be used for monitoring apoptosis and for screening apoptosis inhibitors and activators. An MMP assay was performed according to manufacturer’s instruction with minor modifications. For the experiment, 2 × 10^4^ Caco-2 cells were seeded in each well of a 96-well black plate in a complete culture medium. The cells were incubated overnight at 37 °C under 5% CO_2_. Next, the medium was aspirated and AA in DMEM was added to each well in four repeats and incubated at 37 °C under 5% CO_2_ for 24 h. The final AA concentrations were 0.2, 3.2, 6.4, and 12.5 mM. The negative controls contained only cells in DMEM without AA, while positive controls contained cells in DMEM with 50 µM CCCP. Fluorescence was measured at λ_ex_ 490/ λ_em_ 525 nm. The average green fluorescence was determined as [%] of the negative control, which was assumed to be 100%.

### 3.8. ROS Generation

ROS generation test was performed according to Wu et al. [53]. For the experiment, 2 × 10^4^ Caco-2 cells were seeded in each well of a 96-well black plate in a complete culture medium (for microscopic observations, it was cultured in eight-well Lab-Tek™ Chamber Slides). The cells were incubated overnight at 37 °C under 5% CO_2_. The following day, the medium was aspirated, and cells were washed with PBS. Next, 20 µM DCFH-DA was added to each well. Cells were incubated at 37 °C under 5% CO_2_ for 30 min. After incubation, DCFH-DA was aspirated and cells washed with PBS and AA in DMEM (without FBS) was added to each well (in five repeats) to final concentrations of 0.2, 3.2, and 12.5 mM. The negative controls contained cells in DMEM (without FBS), while positive controls contained cells in DMEM (without FBS) with H_2_O_2_ (500 µM). Cells were incubated at 37 °C under 5% CO_2_ for 6 h. Fluorescence was measured at λ_ex_ 490 and λ_em_ 530 nm. The average DCF fluorescence was determined as [%] of negative control, which was assumed to be 100%. The intracellular fluorescence of cells was observed under fluorescence microscope (Nikon Eclipse Ci H600L, Tokyo, Japan) attached to a digital camera (Nikon Digital Sight DS-U3, Tokyo, Japan) and an imaging software (NIS-elements BR 3.0, Nikon, Tokyo, Japan) at 200× magnification. An increased intensity of intracellular fluorescence was indicative of an increased level of generated ROS.

### 3.9. Apoptosis and Necrosis Detection

Apoptosis was measured by cell death detection ELISA^PLUS^ according to the manufacturer’s instruction. That assay allows quantitative measurement of low molecular weight DNA level that appears after genomic DNA fragmentation catalyzed by endonucleases. The calculated enrichment factor corresponds to mono- and oligonucleosomes present in the cytoplasm fraction at a late stage of apoptosis. Cells were incubated for 24 h with AA final concentrations (0.2, 0.8, 3.2, 6.4, and 12.5 mM). After treatment, cells were lysed and histone-complexed DNA fragments (mono- and oligonucleosomes) present in the cytoplasmic fraction were quantified via an immunoreagent complex. DNA-histone complexes served as the positive control (PC) of apoptosis. Following incubation and washes, the colorimetric solution was added and, after adding the stop solution, the colorimetric signal was measured at 405 and 490 nm. Calculation of the enrichment factor of mono- and oligonucleosomes released into the cytoplasm was performed according to the following formula:

enrichement factor [%]= absorbance of the sample cells/absorbance of the control cells ×100

In order to detect necrosis after cells incubation with compounds, the medium was collected, and the level of DNA fragments released from necrotic cells was determined analogously to the apoptosis measurement.

### 3.10. Giemsa/May–Grünwald Staining

Morphological changes of Caco-2 cells after exposure to AA were observed in eight-well Lab-Tek™ chamber slides. Caco-2 cells were seeded on each well by adding 2.5 × 10^5^ cells/well. The influence of the chosen concentrations of AA (after 24 h exposure, each in two repeats) was 3.2 and 6.4 mM, which were close to the IC_50_ value. After incubation, the medium with AA was gently aspirated, and cells were washed with PBS and fixed with 70% ethanol for 15 min at room temperature. The cells were then stained with the Giemsa/May–Grünwald stain. After staining, the wells were washed with 70% ethanol until no colour remained, and were then air-dried. The morphology of Caco-2 cells was observed at 200× and 400× magnification under a microscope.

### 3.11. DAPI Staining

The nuclear changes in Caco-2 cells in the presence of 3.2 and 6.4 mM (concentration close to IC_50_) AA were observed using eight-well Lab-Tek™ chamber slides (Waltham, MA, USA). DAPI staining was performed according to Giemsa/May–Grünwald staining, each in two repeats. After air-drying, the cells were stained with 1 µg/mL DAPI in the dark. The morphology of cells was observed at 200× magnification under a fluorescent microscope.

### 3.12. AO/PI Double Staining

Caco-2 cells were seeded on each well by adding 1.5 × 10^5^ cells/well of a six-well plate. The tested concentrations of AA were 3.2 and 6.4 mM, after 24 h exposure, each in two repeats. The positive control was 50 µM of CCCP. After that, the medium with AA was gently aspirated; cells were detached, centrifuged (182× *g*, 5 min), and decanted; and the pellet was stained with AO (100 μg/mL) and PI (100 μg/mL) mixture (1:1, *v*/*v*). The morphology of Caco-2 cells was observed at 200× magnification under a fluorescent microscope within five minutes.

### 3.13. SEM

Morphological evaluation of tissues was performed by means of SEM (JEOL JCM-6000, Tokyo, Japan). The samples were prepared according to Osahor et al. [54] with some modifications. Plastic coverslips were sterilized with 70% ethanol and next by UV for 30 min. The coverslips were transferred aseptically into the bottom of a six-well plate and seeded with 2.5 × 10^5^ of Caco-2 cells per well in the complete culture medium. The cells were incubated overnight at 37 °C in 5% CO_2_ to allow them to attach. The medium was changed the following day, and Caco-2 cells were exposed to AA in the concentration of 3.2 mM for 24 h in three repeats. The control sample consisted of cells without AA. After exposition, cells were washed gently with PBS and fixed with 2.5% glutaraldehyde diluted in PBS (30 min, room temperature). Next, cells were dehydrated in alcohol gradient (25%, 40%, 60%, 80%, 90%, and 100%) for 15 min at each concentration. Then, the coverslips were placed in a desiccator and incubated for 24 h. The following day, they were mounted on metal plates and coated with gold particles for 45 s (JEOL JFC-1200 Fine Coater, Tokyo, Japan). All samples were examined in high vacuum at an accelerating voltage of 5.0 kV.

### 3.14. Statistical Analysis

Cytotoxicity and genotoxicity assays data were analyzed using one-way analysis of variance (ANOVA). Additionally, the data were confirmed by Tukey test or Dunnett’s test using GraphPad prism 4.0 software (GraphPad Software, Inc. La Jolla, USA). Significant differences were accepted at * *p* < 0.05. The results were presented as mean ± standard error of the mean (SEM) for PrestoBlue, MMP, ROS generation, and comet assays, and ± standard deviation (SD) for MTT, apoptosis, and necrosis.

## Supplementary Materials

The following are available online: Table S1: Viability of Caco-2 cells in the presence of acrylamide determined by the MTT assay in Caco-2 cells. Each value represents the mean of eight repeats. Results are presented as mean ± standard deviation (SD). * Results significantly different from unexposed control, ANOVA (*p* < 0.05); Table S2: Viability of Caco-2 cells in the presence of acrylamide determined by the PrestoBlue assay in Caco-2 cells. Each value represents the mean of eight repeats. Results are presented as mean ± standard error of the mean (S.E.M.). * Results significantly different from unexposed control, ANOVA (*p* < 0.05).

## References

[1] Bartkiene E., Jakobsone I., Juodeikiene G., Vidmantiene D., Pugajeva I., Bartkevics V. (2013). Study on the reduction of acrylamide in mixed rye bread by fermentation with bacteriocin-like inhibitory substances producing lactic acid bacteria in combination with *Aspergillus niger* glucoamylase. Food Control.

[2] Żyżelewicz D., Oracz J., Krysiak W., Budryn G., Nebesny E. (2017). Effects of various roasting conditions on acrylamide, acrolein and polycyclic aromatic hydrocarbons content in cocoa bean and the derived chocolates. Dry. Technol..

[3] International Agency for Research on Cancer (IARC) (1994). Monographs on the Evaluation of the Carcinogenic Risk of Chemicals to Humans.

[4] Hogervorst J.G., Schouten L.J., Konings E.J., Goldbohm R.A., van den Brandt P.A. (2007). A prospective study of dietary acrylamide intake and the risk of endometrial, ovarian, and breast cancer. Cancer Epidemiol. Biomarkers Prev..

[5] Hogervorst J.G., Schouten L.J., Konings E.J., Goldbohm R.A., van den Brandt P.A. (2008). Dietary acrylamide intake and the risk of renal cell, bladder, and prostate cancer. Am. J. Clin. Nutr..

[6] Żyżelewicz D., Nebesny E., Oracz J. (2010). Acrylamide—Formation, physicochemical and biological properties. Bromat. Chem. Toksykol..

[7] Hogervorst J.G., de Bruijn-Geraets D., Schouten L.J., van Engeland M., de Kok T.M., Goldbohm R.A., van den Brandt P.A., Weijenberg M.P. (2014). Dietary acrylamide intake and the risk of colorectal cancer with specific mutations in KRAS and APC. Carcinogenesis.

[8] Duda-Chodak A., Wajda Ł., Tarko T., Sroka P., Satora P. (2016). A review of the interactions between acrylamide, microorganisms and food components. Food Funct..

[9] Puppel N., Tjaden Z., Fueller F., Marko D. (2005). DNA strand breaking capacity of acrylamide and glycidamide in mammalian cells. Mutat. Res..

[10] CAST–Council for Agricultural Science and Technology (2006). Acrylamide in Food. http://www.cast-science.org/download.cfm?PublicationID=2914&File=f030b1a336fa49052cb068677cf12694a5a4.

[11] Naruszewicz M., Zapolska-Downar D., Kośmider A., Nowicka G., Kozłowska-Wojciechowska M., Vikstrom A.S., Tornqvist M. (2009). Chronic intake of potato chips in humans increases the production of reactive oxygen radicals by leucocytes and increases plasma C-reactive protein: A pilot study. Am. J. Clin. Nutr..

[12] Huang M., Jiao J., Wang J., Xia Z., Zhang Y. (2018). Characterization of acrylamide-induced oxidative stress and cardiovascular toxicity in zebrafish embryos. J. Hazard. Mater..

[13] European Food Safety Authority (EFSA) (2012). Update on acrylamide levels in food from monitoring years 2007 to 2010. Eur. Food Safety Authority J..

[14] FAO/WHO Expert Committee on Food Additives (2011). Evaluations of the Joint FAO/WHO Expert Committee on Food Additives. http://apps.who.int/food-additives-contaminants-jecfa-database/chemical.aspx?chemID=5198.

[15] Koszucka A., Nowak A., Nowak I., Motyl I. (2019). Acrylamide in human diet, its metabolism, toxicity, inactivation and the associated European Union legal regulations in food industry. Crit. Rev. Food Sci. Nutr..

[16] NIH—U.S. National Library of Medicine Acrylamide. https://pubchem.ncbi.nlm.nih.gov/compound/6579#section=UN-Classification.

[17] Jagerstad M., Skog K. (2005). Genotoxicity of heat-processed foods. Mutat. Res..

[18] Chen W., Feng L., Shen Y., Su H., Li Y., Zhuang J., Zhang L., Zheng X. (2013). Myricitrin inhibits acrylamide-mediated cytotoxicity in human Caco-2 cells by preventing oxidative stress. Biomed Res. Int..

[19] Rodríguez-Ramiro I., Martín M.Á., Ramos S., Bravo L., Goya L. (2011). Olive oil hydroxytyrosol reduces toxicity evoked by acrylamide in human Caco-2 cells by preventing oxidative stress. Toxicology.

[20] Qu D., Liu C., Jiang M., Feng L., Chen Y., Han J. (2019). After in vitro digestion, jackfruit flake affords protection against acrylamide-induced oxidative damage. Molecules.

[21] Xu M., McCanna D.J., Sivak J.G. (2015). Use of the viability reagent PrestoBlue in comparison with alamarBlue and MTT to assess the viability of human corneal epithelial cells. J. Pharmacol. Toxicol. Methods.

[22] Sahinturk V., Kacar S., Vejselova D., Kutlu H.M. (2018). Acrylamide exerts its cytotoxicity in NIH/3T3 fibroblast cells by apoptosis. Toxicol. Ind. Health..

[23] Kacar S., Vejselova D., Kutlu H.M., Sahinturk V. (2018). Acrylamide-derived cytotoxic, anti-proliferative, and apoptotic effects on A549 cells. Hum. Exp. Toxicol..

[24] Kacar S., Sahinturk V., Kutlu H.M. (2019). Effect of acrylamide on BEAS-2B normal human lung cells: Cytotoxic, oxidative, apoptotic and morphometric analysis. Acta Histochem..

[25] Chen J.-H., Yang C.-H., Wang Y.-S., Lee J.-G., Chenga C.-H., Chou C.-C. (2013). Acrylamide-induced mitochondria collapse and apoptosis in human astrocytoma cells. Food Chem. Toxicol..

[26] Attoff K., Kertika D., Lundqvist J., Oredsson S., Forsby A. (2016). Acrylamide affects proliferation and differentiation of the neural progenitor cell line C17.2 and the neuroblastoma cell line SH-SY5Y. Toxicol. In Vitro.

[27] Liu Z., Song G., Zou C., Liu G., Wu W., Yuan T., Liu X. (2015). Acrylamide induces mitochondrial dysfunction and apoptosis in BV-2 microglial cells. Free Radic. Biol. Med..

[28] Mallepogu V., Jayasekhar Babu P., Doble M., Suman B., Nagalakshmamma V., Chalapathi P.V., Thyagaraju K. (2017). Effects of acrylamide on cervical cancer (HeLa) cells proliferation and few marker enzymes. Austin J. Biotechnol. Bioeng..

[29] Exon J.H. (2006). A review of the toxicology of acrylamide. J. Toxicol. Environ. Health B Crit. Rev..

[30] Dearfield K.L., Douglas G.R., Ehling U.H., Moore M.M., Sega G.A., Brusick D.J. (1995). Acrylamide: A review of its genotoxicity and an assessment of heritable genetic risk. Mutat. Res..

[31] Hobbs C.A., Jeffrey D., Shepard K., Chepelev N., Friedman M., Marroni D., Recio L. (2016). Differential genotoxicity of acrylamide in the micronucleus and Pig-a gene mutation assays in F344 rats and B6C3F1 mice. Mutagenesis.

[32] Blasiak J., Gloc E., Wozniak K., Czechowska A. (2004). Genotoxicity of acrylamide in human lymphocytes. Chem. Biol. Interact..

[33] Jiang L., Cao J., An Y., Geng C., Qu S., Jiang L., Zhong L. (2007). Genotoxicity of acrylamide in human hepatoma G2 (HepG2) cells. Toxicol. In Vitro.

[34] Recio L., Hobbs C., Caspary W., Witt K.L. (2010). Dose-response assessment of four genotoxic chemicals in a combined mouse and rat micronucleus and comet assay protocol. J. Toxicol. Sci..

[35] Pan X., Zhu Z., Lu L., Wang D., Lu Q., Yan H. (2015). Melatonin attenuates oxidative damage induced by acrylamide in vitro and in vivo. Oxid. Med. Cell. Longevity.

[36] Shimamura Y., Iio M., Urahira T., Masuda S. (2017). Inhibitory effects of Japanese horseradish (Wasabia japonica) on the formation and genotoxicity of a potent carcinogen, acrylamide. J. Sci. Food Agric..

[37] El-Bohi K.M., Moustafa G.G., El-Sharkawi N.I., Sabik L.M.E. (2011). Genotoxic effects of acrylamide in adult male albino rats liver. J. Am. Sci..

[38] Zamani E., Shaki F., Abedian Kenari S., Shokrzadeh M. (2017). Acrylamide induces immunotoxicity through reactive oxygen species production and caspase-dependent apoptosis in mice splenocytes via the mitochondria-dependent signaling pathways. Biomed. Pharmacother..

[39] Seydi E., Rajabi M., Salimi A., Pourahmad J. (2015). Involvement of mitochondrial-mediated caspase-3 activation and lysosomal labilization in acrylamide-induced liver toxicity. Toxicol. Environ. Chem..

[40] Chen W., Su H., Xu Y., Jin C. (2017). In vitro gastrointestinal digestion promotes the protective effect of blackberry extract against acrylamide-induced oxidative stress. Sci. Rep..

[41] Ghorbel I., Elwej A., Fendri N., Mnif H., Jamoussi K., Boudawara T., Grati Kamoun Z., Zeghal N. (2017). Olive oil abrogates acrylamide induced nephrotoxicity by modulating biochemical and histological changes in rats. Ren. Fail..

[42] Erdemli M.E., Aksungur Z., Gul M., Yigitcan B., Bag H.G., Altinoz E., Turkoz Y. (2019). The effects of acrylamide and vitamin E on kidneys in pregnancy: An experimental study. J. Matern. Fetal Neonatal. Med..

[43] Koszucka A., Nowak A. (2019). Thermal processing food-related toxicants: A Review. Crit. Rev. Food Sci. Nutr..

[44] Pan X., Yan D., Wang D.Y., Wu X., Zhao W., Lu Q., Yan H.W. (2016). Mitochondrion-mediated apoptosis induced by acrylamide is regulated by a balance between Nrf2 antioxidant and MAPK signaling pathways in PC12 cells. Mol. Neurobiol..

[45] Pan X., Wu X., Yan D., Peng C., Rao C., Yan H. (2018). Acrylamide-induced oxidative stress and inflammatory response are alleviated by N-acetylcysteine in PC12 cells: Involvement of the crosstalk between Nrf2 and NF-κB pathways regulated by MAPKs. Toxicol. Lett..

[46] Sun J., Li M., Zou F., Bai S., Jiang X., Tian L., Ou S., Jiao R., Bai W. (2018). Protection of cyanidin-3-O -glucoside against acrylamide- and glycidamide-induced reproductive toxicity in leydig cells. Food Chem. Toxicol..

[47] Baskić D., Popović S., Ristić P., Arsenijević N.N. (2006). Analysis of cycloheximide-induced apoptosis in human leukocytes: Fluorescence microscopy using annexin V/propidium iodide versus acridin orange-ethidium bromide. Cell Biol. Int..

[48] Jiang G., Zhang L., Wang H., Chen Q., Wu X., Yan X., Chen Y., Xie M. (2018). Protective effects of a *Ganoderma atrum* polysaccharide against acrylamide induced oxidative damage via a mitochondria mediated intrinsic apoptotic pathway in IEC-6 cells. Food Func..

[49] Nowak A., Sójka M., Klewicka E., Lipińska L., Klewicki R., Kołodziejczyk K. (2017). Ellagitannins from *Rubus idaeus* L. Exert geno- and cytotoxic effects against human colon adenocarcinoma cell line Caco-2. J. Agric. Food Chem..

[50] Nowak A., Bakuła T., Matusiak K., Gałęcki R., Borowski S., Gutarowska B. (2017). Odorous compounds from poultry manure induce DNA Damage, nuclear changes, and decrease cell membrane integrity in chicken liver hepatocellular carcinoma cells. Int. J. Environ. Res. Public Health.

[51] OECD Guidelines for the Testing of Chemicals, Section 4 Test No. 442D: In Vitro Skin Sensitisation ARE-Nrf2 Luciferase Test Method. https://ntp.niehs.nih.gov/iccvam/suppdocs/feddocs/oecd/oecd-tg442d-508.pdf.

[52] Nowak A., Czyżowska A., Stańczyk M. (2015). Protective activity of probiotic bacteria against 2-amino-3-methyl-3H-imidazo[4,5-f]quinoline (IQ) and 2-amino-1-methyl-6-phenyl-1H-imidazo[4,5-b]pyridine (PhIP)—An in vitro study. Food Addit. Contam. Part. A.

[53] Wu D., Yotnda P. (2011). Production and detection of reactive oxygen species (ROS) in cancers. J. Vis. Exp..

[54] Osahor A., Deekonda K., Lee C.W., Sim E.U., Radu A., Narayanan K. (2017). Rapid preparation of adherent mammalian cells for basic scanning electron microscopy (SEM) analysis. Anal. Biochem..

