# Supplementary material for: Acrylamide Decreases Cell Viability, and Provides Oxidative Stress, DNA Damage, and Apoptosis in Human Colon Adenocarcinoma Cell Line Caco-2
Source: Molecules. 2020 Jan 16;25(2):368. doi: 10.3390/molecules25020368 (PMC7024287; doi:10.3390/molecules25020368)
Supplement: Supplementary file 1 [file molecules-25-00368-s001.pdf]

Supporting Information

Table S1. Viability of Caco-2 cells in the presence of acrylamide determined by the MTT assay in Caco-2 cells after 24 h, 48 h and 72 h exposure. Each value represents the mean of eight repeats. Results are presented as mean ± standard deviation (SD). * Results significantly different from unexposed control, ANOVA ($p < 0.05$).

**Acrylamide concentration [mM]**	**Viability of Caco-2 cells [%] ± SD**		
	**24 h**	**48 h**	**72 h**
0.2	114.0 ± 0.7	122.6 ± 1.6	105.4 ± 1.2
0.4	123.8 ± 0.4	113.8 ± 1.9	90.9 ± 1.1
0.8	114.8 ± 0.5	98.1 ± 1.6	45.1 ± 1.4*
1.6	113.4 ± 0.5	90.1 ± 1.3	26.8 ± 0.2*
3.2	98.3 ± 1.7	24.5 ± 0.5*	10.9 ± 0.8*
6.4	43.9 ± 0.8*	11.3 ± 0.1*	7.6 ± 0.3*
12.5	12.4 ± 0.2*	14.2 ± 0.3*	5.4 ± 0.3*
25	16.0 ± 0.1*	18.9 ± 0.2*	8.2 ± 0.2*
50	14.2 ± 0.1*	16.0 ± 0.1*	5.6 ± 0.1*

Table S2. Viability of Caco-2 cells in the presence of acrylamide determined by the PrestoBlue assay. Each value represents the mean of eight repeats. Results are presented as mean ± standard error of the mean (S.E.M.). * Results significantly different from unexposed control, ANOVA ($p < 0.05$).

**Acrylamide concentration [mM]**	**Viability of Caco-2 cells [%] ± SD**		
	**24 h**	**48 h**	**72 h**
0.2	101.9 ± 7.4	106.1 ± 2.2	121.0 ± 2.5*
0.4	98.7 ± 9.7	97.7 ± 16.2	107.7 ± 5.9
0.8	99.6 ± 4.8	96.6 ± 3.3	98.3 ± 14.9
1.6	88.4 ± 11.6	77.0 ± 16.9*	81.9 ± 13.6*
3.2	85.9 ± 9.8	57.2 ± 21.5*	32.7 ± 3.3*
6.4	62.1 ± 8.9*	27.8 ± 1.5*	26.3 ± 1.0*
12.5	33.4 ± 16.3*	23.7 ± 0.8*	22.9 ± 0.8*
25	25.1 ± 1.7*	20.6 ± 0.6*	20.0 ± 0.6*
50	21.6 ± 0.9*	17.8 ± 0.5*	17.8 ± 0.6*
